# Comparative prognosis and risk assessment in gallbladder neuroendocrine neoplasms versus adenocarcinomas

**DOI:** 10.3389/fendo.2024.1326112

**Published:** 2024-02-08

**Authors:** Zhi-Hao Zhao, Yu Huang, Chao Jiang, Guo-Yue Lv, Meng Wang

**Affiliations:** Department of Hepatobiliary and Pancreatic Surgery, General Surgery Center, The First Hospital of Jilin University, Changchun, Jilin, China

**Keywords:** gallbladder neuroendocrine neoplasms, gallbladder adenocarcinomas, propensity score matching, prognosis, nomogram, overall survival

## Abstract

**Background:**

Gallbladder neuroendocrine neoplasms (GB-NENs) are a rare malignant disease, with most cases diagnosed at advanced stages, often resulting in poor prognosis. However, studies regarding the prognosis of this condition and its comparison with gallbladder adenocarcinomas (GB-ADCs) have yet to yield convincing conclusions.

**Methods:**

We extracted cases of GB-NENs and GB-ADCs from the Surveillance, Epidemiology, and End Results (SEER) database in the United States. Firstly, we corrected differences in clinical characteristics between the two groups using propensity score matching (PSM). Subsequently, we visualized and compared the survival outcomes of the two groups using the Kaplan-Meier method. Next, we employed the least absolute shrinkage and selection operator (LASSO) regression and Cox regression to identify prognostic factors for GB-NENs and constructed two nomograms for predicting prognosis. These nomograms were validated with an internal validation dataset from the SEER database and an external validation dataset from a hospital. Finally, we categorized patients into high-risk and low-risk groups based on their overall survival (OS) scores.

**Results:**

A total of 7,105 patients were enrolled in the study, comprising 287 GB-NENs patients and, 6,818 GB-ADCs patients. There were substantial differences in clinical characteristics between patients, and GB-NENs exhibited a significantly better prognosis. Even after balancing these differences using PSM, the superior prognosis of GB-NENs remained evident. Independent prognostic factors selected through LASSO and Cox regression were age, histology type, first primary malignancy, tumor size, and surgery. Two nomograms for prognosis were developed based on these factors, and their performance was verified from three perspectives: discrimination, calibration, and clinical applicability using training, internal validation, and external validation datasets, all of which exhibited excellent validation results. Using a cutoff value of 166.5 for the OS nomogram score, patient mortality risk can be identified effectively.

**Conclusion:**

Patients with GB-NENs have a better overall prognosis compared to those with GB-ADCs. Nomograms for GB-NENs prognosis have been effectively established and validated, making them a valuable tool for assessing the risk of mortality in clinical practice.

## 1 Introduction

Neuroendocrine neoplasms (NENs) are rare and heterogeneous malignancies originating from neuroendocrine cells, which can be found in almost all organs and tissues of the human body (1, 2). However, these neoplasms are predominantly identified in the gastrointestinal and respiratory tracts (3). NENs in the gallbladder are even rarer, comprising only 0.5% of all NENs and 2% of all gallbladder neoplasms, as reported previously (4, 5). In recent years, with increased awareness of the disease and advancements in diagnostic methods, the detection rate of gallbladder neuroendocrine neoplasms (GB-NENs) has been gradually rising (3, 6).

Despite rapid progress in the understanding and treatment of NENs, research remains limited due to their rarity. Unlike neoplasms in other locations, GB-NENs typically do not present with symptoms and are often diagnosed at advanced stages (7). Summarizing published cases and clinical studies indicates that the management of GB-NENs varies widely, ranging from simple cholecystectomy to radical resection, along with adjuvant therapies such as radiation and chemotherapy (7–13). Besides, radical surgery is generally considered the primary approach to treating this disease, and the efficacy of adjuvant treatments has not been fully established (14–16). Current treatment strategies do not effectively prevent adverse outcomes for patients.

Furthermore, recent research reports conflicting results regarding the prognosis of GB-NENs compared to gallbladder adenocarcinomas (GB-ADCs) (7, 17–19). In current clinical practice, our understanding of the overall prognosis of GB-NENs is limited, relying on a small number of case analyses, which are often unreliable and subject to significant error.

In this study, to achieve a more robust analysis, we extracted cases of GB-NENs and GB-ADCs from the Surveillance, Epidemiology, and End Results (SEER) database. We balanced the clinical characteristics of both diseases using propensity score matching (PSM) and then compared their prognoses, resulting in more reliable research findings. Additionally, to enhance our understanding of the disease, we constructed two nomograms for GB-NENs, which are a reliable and visual statistical predictive model. By analyzing crucial prognostic indicators, it accurately stratifies patients’ risk. To assess the nomogram’s performance, we conducted validation of prognostic predictions and risk stratification using both the internal validation set from the SEER database and the external validation set from our medical institution.

## 2 Materials and methods

### 2.1 Data source and case selection

Patient data for individuals with GB-NENs and GB-ADCs were sourced from the SEER database of the National Cancer Institute (NCI) in the United States. The SEER database is publicly accessible and contains information on millions of cancer patients from various regions across the United States (20). Since it is an anonymized database, ethical approval was not required for its use. Additionally, we included GB-NENs patients who had been treated at the First Hospital of Jilin University between, 2010 and, 2020. Given the retrospective nature of the study and the concealment of patients’ private information, the study obtained only verbal informed consent from patients and was determined to be exempt from relevant ethical approval. The implementation of this study fully adhered to the requirements of the Helsinki Declaration of, 1964.

### 2.2 Clinical information acquisition and screening criteria

**Figure 1:**
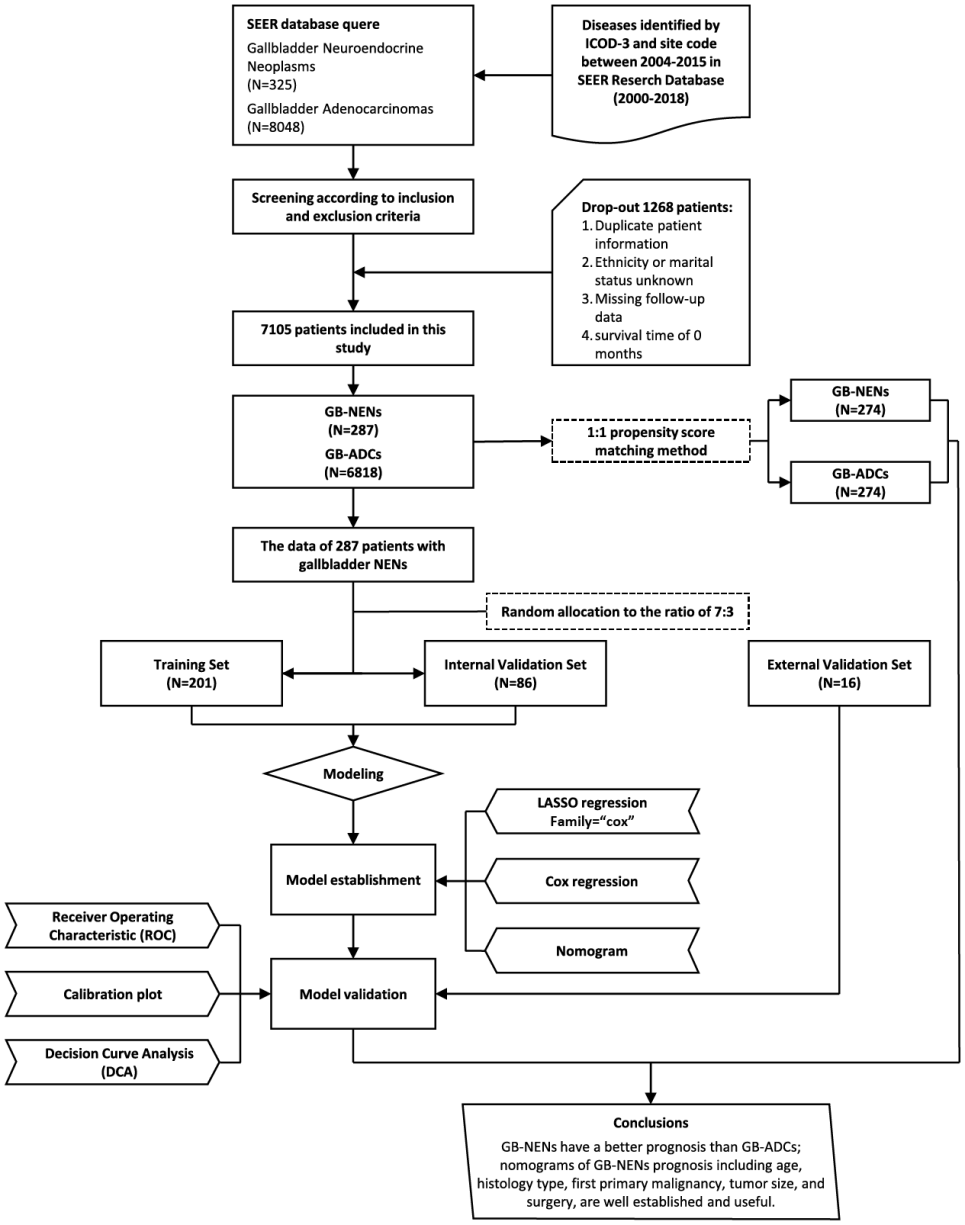
Flowchart of the study.

We extracted patient data in the case database about 18 SEER registries, spanned from, 2000 to, 2018, using SEER*Stat 8.4.2 software. The inclusion criteria detected cases diagnosed between, 2004 and, 2015 with the site code C23.9, encompassing histology subtypes, 8140/3 for ADCs and, 8013/3, 8041/3, 8140/3, 8240/3, 8244/3, 8246/3, and, 8249/3 for NENs. The exclusion criteria included cases with duplicate patient IDs, missing data regarding race or marital status, missing follow-up information, and cases with a survival time of 0. The selection process is illustrated in **Figure 1**. This led to the final inclusion of 287 GB-NENs patients and, 6,818 GB-ADCs patients.

We systematically gathered demographic variables of patients, crucial prognostic factors presented in prior studies, as well as variables deemed significant through empirical clinical experience, alongside survival information. The collected data includes gender, age, race, marital status at diagnosis, year of diagnosis, histology type, first malignant primary indicator, tumor size, pathological grade, surgery on primary site, lymph node surgery, radiation, chemotherapy, survival months, cause-specific death, and other cause of death. Other important data such as TNM information were omitted due to much missing data in GB-NENs patients. The primary endpoints of this study were all-cause death and disease-specific death.

In addition, we gathered data on 11 patients with surgical resection and 5 patients who did not undergo surgery at the First Hospital of Jilin University during the period from, 2010 to, 2020. The medical records and follow-up information were fully accessible, with the final follow-up date being June 30, 2023. The outcome was defined as all-cause death. Exclusion criteria and required variables were consistent with the standards above.

### 2.3 Data preprocessing before analysis

The collected variables were processed as follows: gender, categorized as male or female; age, stratified into age groups below 60 years, 60-80 years, and above 80 years; race, with options of white, black, and other, which included Asian or Pacific Islander and American Indian/Alaska Native; marital status, divided into single, married, and other, encompassing categories divorced, separated and widowed; year of diagnosis, distinguished as before, 2010 and, 2010 or later; first primary malignancy, indicated as yes or no; tumor size, categorized as below 50 mm, above 50 mm, or unknown; pathological grade, classified as I/II, III/IV, or unknown; and surgery on primary site, lymph node surgery, radiation and chemotherapy, all recorded as either yes or no. Survival time, measured in months, was reported in two forms: Overall Survival (OS), signifying the time from disease diagnosis to death from any cause, and Cancer-Specific Survival (CSS), signifying the time from disease diagnosis to death due specifically to the disease and not other causes.

### 2.4 PSM and survival analysis

We employed Chi-squared tests or Fisher’s exact tests to assess differences in patient characteristics between GB-NENs and GB-ADCs cohorts. Subsequently, we applied PSM using the “Matchit” R package, with the following fundamental settings: 1:1 matching, nearest-neighbor matching method, and a caliper width of 0.05 (21). To compare the prognosis between the two groups, we utilized the Kaplan-Meier method to visualize the survival rate changes for each patient group, followed by log-rank tests to assess differences in both OS and CSS.

### 2.5 Development and validation of prognostic model

We initially selected 287 GB-NENs patients from the SEER database. Using randomly generated numbers in R, we allocated them into a training set (N=201) and an internal validation set (N=86) at a 7:3 ratio. We applied Chi-squared tests or Fisher’s exact tests to assess differences between these two groups. Additionally, we collected data from the hospital to create an external validation set (N=16). The training set was used to develop nomograms for OS and CSS. Subsequently, we validated these nomograms using the internal and external validation sets. We further stratified OS nomogram scores using X-tile software for risk stratification. The primary analytical process is illustrated in **Figure 1**.

Variable selection was executed through least absolute shrinkage and selection operator (LASSO) regression and multivariable Cox regression. LASSO regression effectively mitigates issues of multicollinearity among variables, while Cox regression, under the proportional hazards assumption, addresses the magnitude of the effects of multiple covariates in survival analysis. This approach was employed to further identify variables associated with prognosis (22). Finally, two multivariate Cox risk models were utilized to estimate OS and CSS, and nomograms were created for both. Nomogram visually represents the associations between variables included in the model using proportional line segments. Each patient was assigned some scores based on the contribution of each variable to the outcome (i.e., the magnitude of regression coefficients). These individual scores were then aggregated to obtain a total score, transformed into a function of the probability of event occurrence, thereby expressing the predicted probability of the outcome.

We assessed model performance using the concordance index (C-index), receiver operating characteristic (ROC) curve, and the area under the receiver operating characteristic curve (AUC) at various time points (23). The C-index evaluates overall discriminatory ability, with values above 0.7 indicating good discrimination (24). AUC values are positively correlated with predictive ability, with a range of 0.5 to 1, where 0.5 suggests no predictive ability and 1 indicates perfect prediction. We estimated AUC values at 6, 12, 36, and 60 months. Calibration curves demonstrate the relationship between observed event frequencies and predicted probabilities. A 45-degree calibration curve indicates perfect alignment between predicted and observed probabilities. Deviation from this line represents predictive bias (25). We plotted calibration curves at 6, 12, 36, and 60 months. Additionally, we employed decision curve analysis (DCA) to assess net benefit. DCA curves include two reference lines, one for giving all treatments and one for giving no treatment. The model’s curve is compared to these reference lines, with greater separation indicating improved net benefit (26).

Finally, we calculated the hazard scores for OS using prognostic nomogram and stratified patients into high-risk and low-risk groups based on these scores. We then used Kaplan-Meier methods and log-rank tests to compare survival outcomes between different groups. All analyses were conducted using R version 4.3.1 during the period from September 1, 2023, to September 30, 2023, and all p-values were based on two-tailed tests, with statistical significance set at p<0.05.

## 3 Results

### 3.1 Comparison of the clinical characteristics of GB-NENs and GB-ADCs

After the selection process, a total of 7,105 patients were included in this study from the SEER database, comprising 287 GB-NENs patients and 6,818 GB-ADCs patients (see **Figure 1**). In the original data, notable imbalances existed in some clinical characteristics between the two groups. For instance, concerning demographic features, the GB-NENs group had a higher proportion of patients below 60 years old compared to GB-ADCs (24% vs. 38%, p<0.001). Married status was more represented in GB-NENs compared to other marital statuses (62.7% vs. 52.4%, p=0.003). Regarding neoplasm information, GB-NENs patients had a higher percentage of neoplasms measuring less than 50 mm (50.9% vs. 43.3%, p<0.001), but the tumor differentiation in GB-NENs was comparatively poorer, with a lower proportion of grade I/II neoplasms (14.6% vs. 45.8%, p<0.001). In terms of treatment, a lower percentage of GB-NENs patients underwent lymph node surgery (24.4% vs. 33.4%, p=0.002), and fewer of them received radiation therapy (11.1% vs. 15.7%, p=0.044). On the other hand, some characteristics such as gender, race, year of diagnosis, first primary malignancy, surgery, and chemotherapy did not exhibit significant differences between the two groups (p>0.05).

### 3.2 PSM and survival analysis

**Figure 2:**
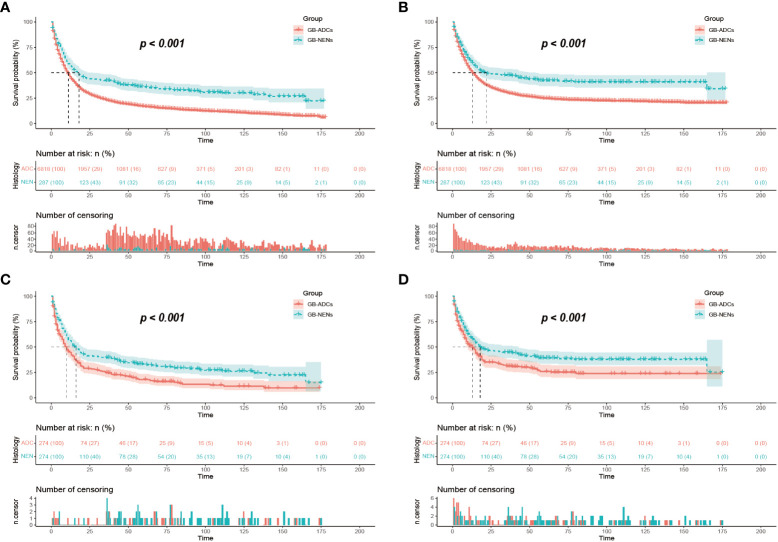
Survival outcomes before and after PSM. OS **(A)** and CSS **(B)** of GB-NENs and GB-ADCs patients before PSM; OS **(C)** and CSS **(D)** of GB-NENs and GB-ADCs patients after PSM. Log-rank tests were used to generate the P-values. PSM, propensity score matching; OS, overall survival; CSS, cancer-specific survival; GB-NENs, gallbladder neuroendocrine neoplasms; GB-ADCs, gallbladder adenocarcinomas.

After performing PSM to balance the differences between GB-NENs and GB-ADCs groups (with all standard differences less than 0.1), a total of 548 patients (274 GB-NENs and 274 GB-ADCs) were included in the final analysis (see **Table 1**). Survival analyses were conducted for both GB-NENs and GB-ADCs groups before and after PSM. Before PSM, a total of 6,105 patients were included, and the median OS time for GB-NENs and GB-ADCs was 18 months and 11 months, respectively. The median CSS time for GB-NENs and GB-ADCs was 22 months and 13 months, respectively. GB-NENs had a better prognosis, with 1, 3, and 5-year OS rates of 58.2% vs. 46.1%, 41.5% vs. 23.0%, and 35.8% vs. 17.0%, respectively. Similarly, the 1, 3, and 5-year CSS rates were 61.6% vs. 51.9%, 47.0% vs. 30.1%, and 41.7% vs. 24.8%, respectively. These differences were statistically significant (p<0.001) (see **Figures 2A**, **B**). After PSM, the median OS time for GB-NENs and GB-ADCs was 16 months and 10 months, respectively. The median CSS time was 18 months for GB-NENs and 13 months for GB-ADCs. GB-NENs still exhibited a better prognosis, with 1, 3, and 5-year OS rates of 56.2% vs. 44.8%, 38.7% vs. 24.4%, and 32.6% vs. 17.0%, respectively. The 1, 3, and 5-year CSS rates were 59.7% vs. 49.0%, 44.3% vs. 30.7%, and 38.6% vs. 25.2%, respectively. These differences remained statistically significant (p<0.001) (see **Figures 2C**, **D**). Therefore, after adjusting for demographic factors, tumor characteristics, and treatment methods through PSM, GB-NENs continued to demonstrate a superior prognosis compared to GB-ADCs, consistent with the initial findings.

**Table 1:** Clinical characteristics of GB-NENs and GB-ADCs before and after PSM.

Subject	Before PSM		P-value	After PSM		P-value
Characteristic	GB-ADCs	GB-NENs		GB-ADCs	GB-NENs	
	N (%)	N (%)		N (%)	N (%)	
All	6818	287		274	274	
**Gender**			0.076			0.721
Female	4766 (69.9)	186 (64.8)		175 (63.9)	180 (65.7)	
Male	2052 (30.1)	101 (35.2)		99 (36.1)	94 (34.3)	
**Race**			0.215			0.753
White	5232 (76.7)	223 (77.7)		211 (77.0)	214 (78.1)	
Black	840 (12.3)	41 (14.3)		35 (12.8)	37 (13.5)	
Otder	746 (10.9)	23 (8.01)		28 (10.2)	23 (8.39)	
**Age, y**			**<0.001**			0.983
≤60	1639 (24.0)	109 (38.0)		99 (36.1)	97 (35.4)	
60~80	3762 (55.2)	139 (48.4)		136 (49.6)	138 (50.4)	
>80	1417 (20.8)	39 (13.6)		39 (14.2)	39 (14.2)	
**Marital status**			**0.003**			0.611
Single	915 (13.4)	31 (10.8)		23 (8.39)	29 (10.6)	
Married	3576 (52.4)	180 (62.7)		180 (65.7)	171 (62.4)	
Otder	2327 (34.1)	76 (26.5)		71 (25.9)	74 (27.0)	
**Year of diagnosis**			0.291			>0.999
<2010	3103 (45.5)	121 (42.2)		112 (40.9)	113 (41.2)	
≥2010	3715 (54.5)	166 (57.8)		162 (59.1)	161 (58.8)	
**First primary malignancy**			0.118			0.701
No	1025 (15.0)	33 (11.5)		37 (13.5)	33 (12.0)	
Yes	5793 (85.0)	254 (88.5)		237 (86.5)	241 (88.0)	
**Tumor size, mm**			**<0.001**			0.617
≤50	2951 (43.3)	146 (50.9)		131 (47.8)	133 (48.5)	
>50	815 (12.0)	50 (17.4)		43 (15.7)	50 (18.2)	
Unknown	3052 (44.8)	91 (31.7)		100 (36.5)	91 (33.2)	
**Patdological grade**			**<0.001**			0.992
I/II	3123 (45.8)	42 (14.6)		43 (15.7)	42 (15.3)	
III/IV	2093 (30.7)	112 (39.0)		112 (40.9)	112 (40.9)	
unknown	1602 (23.5)	133 (46.3)		119 (43.4)	120 (43.8)	
**Surgery on primary site**			0.196			0.643
No/Unknown	1819 (26.7)	87 (30.3)		81 (29.6)	87 (31.8)	
Yes	4999 (73.3)	200 (69.7)		193 (70.4)	187 (68.2)	
**Lymph node surgery**			**0.002**			0.769
No/Unknown	4542 (66.6)	217 (75.6)		202 (73.7)	206 (75.2)	
Yes	2276 (33.4)	70 (24.4)		72 (26.3)	68 (24.8)	
**Radiation**			**0.044**			0.786
No/Unknown	5746 (84.3)	255 (88.9)		245 (89.4)	242 (88.3)	
Yes	1072 (15.7)	32 (11.1)		29 (10.6)	32 (11.7)	
**Chemotderapy**			0.864			0.664
No/Unknown	4063 (59.6)	173 (60.3)		166 (60.6)	160 (58.4)	
Yes	2755 (40.4)	114 (39.7)		108 (39.4)	114 (41.6)	

The bold values indicate P<0.05.

PSM, propensity score matching; GB-NENs, gallbladder neuroendocrine neoplasms; GB-ADCs, gallbladder adenocarcinomas.

### 3.3 Identification of prognostic factors

**Figure 3:**
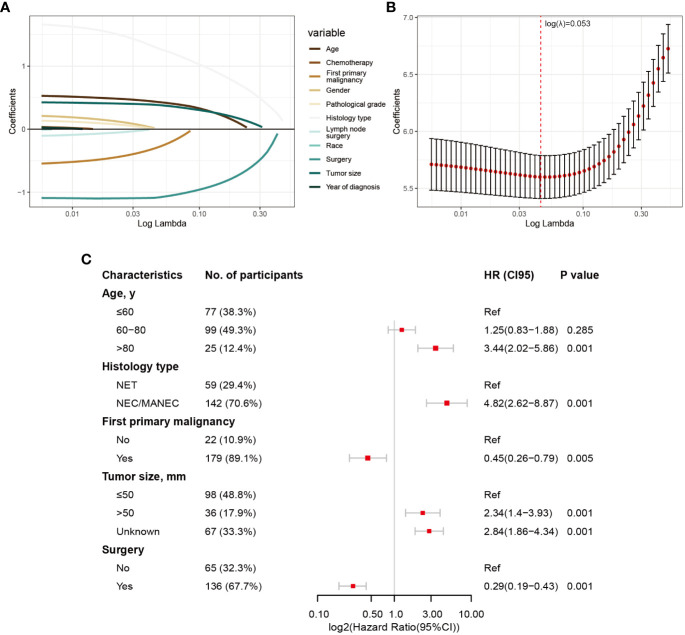
Risk factor selection for OS in GB-NENs patients. **(A)** changes in coefficients in LASSO regression; **(B)** ten-fold cross-validation for LASSO regression; **(C)** multivariable Cox regression. OS, Overall Survival; GB-NENs, gallbladder neuroendocrine neoplasms; LASSO, the least absolute shrinkage and selection operator. NET, neuroendocrine tumor; NEC, neuroendocrine carcinoma; MANEC, mixed adenoneuroendocrine carcinoma.

**Figure 4:**
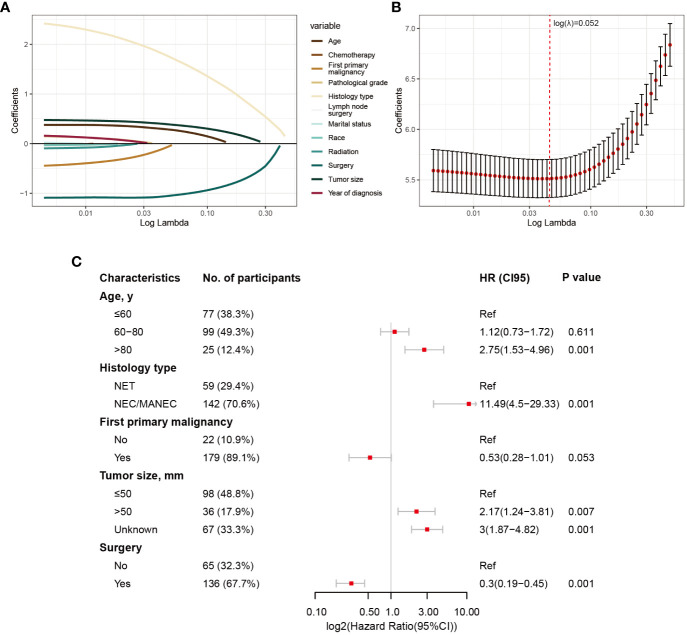
Risk factor selection for CSS in GB-NENs patients. **(A)** changes in coefficients in LASSO regression; **(B)** ten-fold cross-validation for LASSO regression; **(C)** multivariable Cox regression. CSS, cancer-specific survival; GB-NENs, gallbladder neuroendocrine neoplasms; LASSO, the least absolute shrinkage and selection operator; NET, neuroendocrine tumor; NEC, neuroendocrine carcinoma; MANEC, mixed adenoneuroendocrine carcinoma.

The division results of the training set and internal validation set are shown in **Table 2**, with no significant differences in baseline variables. We initially performed LASSO regression to preliminarily select potential clinical features that could affect the prognosis of GB-NENs patients. As the penalty coefficient log(λ) increased, the regression coefficients of variables gradually approached zero. After 10-fold cross-validation, the optimal log(λ) values for OS (**Figures 3A**, **B**) and CSS (**Figures 4A**, **B**) were found to be 0.053 and 0.052, respectively. Twelve variables were reduced to seven (gender, race, marital status, year of diagnosis, histology type, lymph node surgery, radiation, and chemotherapy). Ultimately, we identified age, histology type, first primary malignancy, tumor size, and surgery as significant prognostic factors for OS and CSS in GB-NENs patients.

**Table 2:** Clinical characteristics of the training set and internal validation set in GB-NENs.

Characteristic	All	Training Set	Internal Validation Set	P-value
	N(%)	N(%)	N(%)	
All	287	201	86	
Histology type				0.993
NETs	85 (29.6)	59 (29.4)	26 (30.2)	
NECs/MANECs	202 (70.4)	142 (70.6)	60 (69.8)	
**Gender**				>0.999
Female	186 (64.8)	130 (64.7)	56 (65.1)	
Male	101 (35.2)	71 (35.3)	30 (34.9)	
**Race**				0.660
White	223 (77.7)	155 (77.1)	68 (79.1)	
Black	41 (14.3)	28 (13.9)	13 (15.1)	
Otder	23 (8.0)	18 (8.96)	5 (5.81)	
**Age, y**				0.682
≤60	109 (38.0)	77 (38.3)	32 (37.2)	
60~80	139 (48.4)	99 (49.3)	40 (46.5)	
>80	39 (13.6)	25 (12.4)	14 (16.3)	
**Marital status**				0.944
Single	31 (10.8)	21 (10.4)	10 (11.6)	
Married	180 (62.7)	126 (62.7)	54 (62.8)	
Otder	76 (26.5)	54 (26.9)	22 (25.6)	
**Year of diagnosis**				0.746
<2010	121 (42.2)	83 (41.3)	38 (44.2)	
≥2010	166 (57.8)	118 (58.7)	48 (55.8)	
**First primary malignancy**				0.805
No	33 (11.5)	22 (10.9)	11 (12.8)	
Yes	254 (88.5)	179 (89.1)	75 (87.2)	
**Tumor size, mm**				0.538
≤50	146 (50.9)	98 (48.8)	48 (55.8)	
>50	50 (17.4)	36 (17.9)	14 (16.3)	
Unknown	91 (31.7)	67 (33.3)	24 (27.9)	
**Patdological grade**				0.976
I/II	42 (14.6)	30 (14.9)	12 (14.0)	
III/IV	112 (39.0)	78 (38.8)	34 (39.5)	
unknown	133 (46.4)	93 (46.3)	40 (46.5)	
**Surgery on primary site**				0.317
No/Unkown	87 (30.3)	65 (32.3)	22 (25.6)	
Yes	200 (69.7)	136 (67.7)	64 (74.4)	
**Lymph node surgery**				>0.999
No/Unkown	217 (75.6)	152 (75.6)	65 (75.6)	
Yes	70 (24.4)	49 (24.4)	21 (24.4)	
**Radiation**				0.392
No/Unkown	255 (88.9)	176 (87.6)	79 (91.9)	
Yes	32 (11.1)	25 (12.4)	7 (8.14)	
**Chemotderapy**				>0.999
No/Unkown	173 (60.3)	121 (60.2)	52 (60.5)	
Yes	114 (39.7)	80 (39.8)	34 (39.5)	

NETs, neuroendocrine tumors; NECs, neuroendocrine carcinomas; MANECs, mixed adenoneuroendocrine carcinomas.

These LASSO-selected variables were then included in a multivariable Cox regression analysis. The results revealed that older age (‘>80 y’ vs. ‘≤60 y’, HR (95% CI) 3.44 (2.02–5.86), p<0.001), poorer histology type (‘NEC/MANEC’ vs. ‘NET’, HR (95% CI) 4.82 (2.62–8.87), p<0.001), and larger tumor size (‘>50 mm’ vs. ‘≤50 mm’, HR (95% CI) 2.34 (1.4–3.93), p<0.001) were independent risk factors for OS in GB-NENs patients. Additionally, first primary malignancy (‘Yes’ vs. ‘No’, HR (95% CI) 0.45 (0.26–0.79), p =0.005) and surgery (‘Yes’ vs. ‘No’, HR (95% CI) 0.29 (0.19–0.43), p<0.001) were identified as independent protective factors for OS (see **Figure 3C**).

Simultaneously, older age (‘>80 y’ vs. ‘≤60 y’, HR (95% CI) 2.75 (1.53–4.96), p<0.001), poorer histology type (‘NEC/MANEC’ vs. ‘NET’, HR (95% CI) 11.49 (4.5–29.33), p<0.001), and larger tumor size (‘>50 mm’ vs. ‘≤50 mm’, HR (95% CI) 2.17 (1.24–3.81), p=0.007) were found to be independent risk factors for CSS in GB-NENs patients. Furthermore, surgery (‘Yes’ vs. ‘No’, HR (95% CI) 0.3 (0.19–0.45), p<0.001) was established as an independent protective factor for CSS (see **Figure 4C**).

### 3.4 Construction and validation of nomograms for predicting OS and CSS

**Figure 5:**
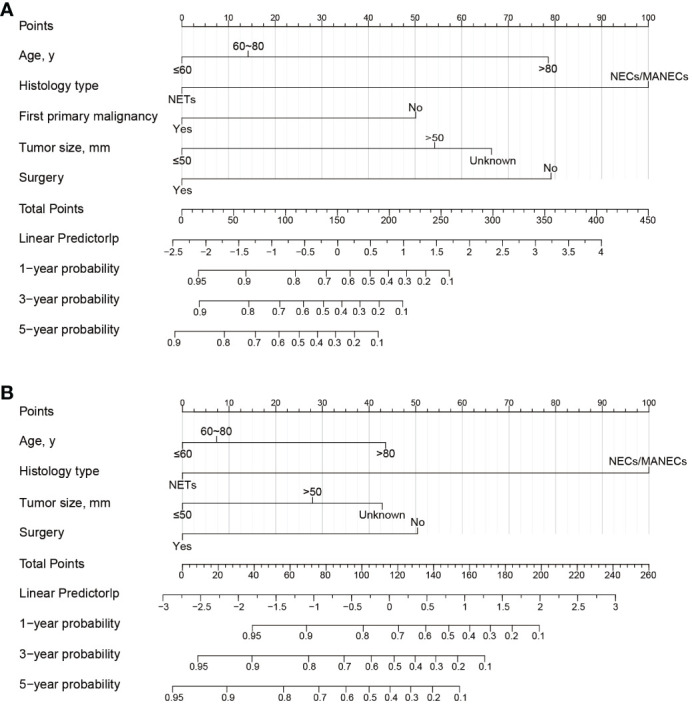
Nomograms for predicting prognosis in GB-NENs patients. **(A)** OS; **(B)**, CSS. GB-NENs, gallbladder neuroendocrine neoplasms; OS, overall survival; CSS, cancer-specific survival. NETs, neuroendocrine tumors; NECs, neuroendocrine carcinomas; MANECs, mixed adenoneuroendocrine carcinomas.

**Figure 6:**
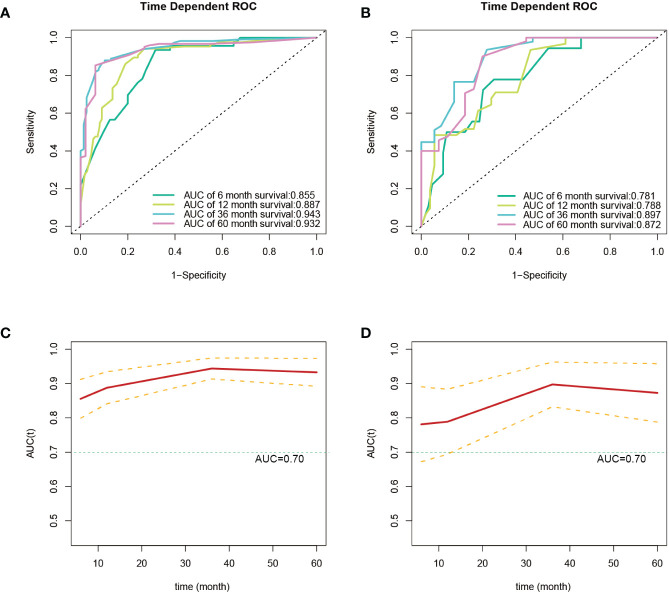
ROC and AUC curves for predicting OS in GB-NENs patients. **(A)** ROC curve for the training set; **(B)** ROC curve for the internal validation set; **(C)** AUC curve for the training set; **(D)** AUC curve for the internal validation set. ROC, receiver operating characteristic; AUC, area under the receiver operating characteristic curve; OS, overall survival; GB-NENs, gallbladder neuroendocrine neoplasms.

**Figure 7:**
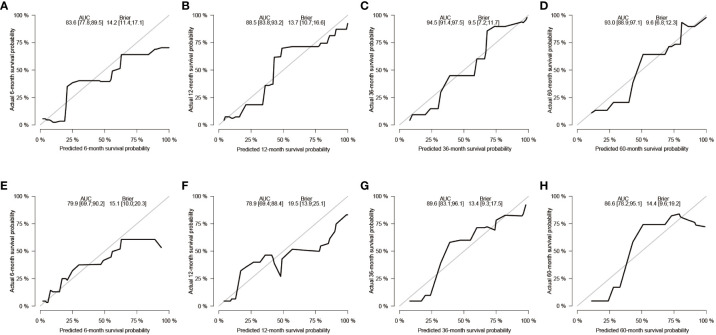
Calibration curves for predicting OS in GB-NENs patients. **(A–D)** calibration curves for 6, 12, 36, and 60 months in the training set; **(E–H)** calibration curves for 6, 12, 36, and 60 months in the internal validation set. OS, overall survival; GB-NENs, gallbladder neuroendocrine neoplasms.

After the aforementioned selection, we used data from the training set to create an OS predictive nomogram for GB-NENs. It incorporated five prognostic factors, including age, histology type, first primary malignancy, tumor size, and surgery, as shown in **Figure 5A**. In the internal validation, the C-index of the nomogram was calculated to be 0.820 (95% CI, 0.785-0.855), indicating good discriminative ability. Additionally, time-ROC analysis was conducted to assess the predictive performance of the model at four different time points (6 months, 12 months, 36 months, and 60 months). The AUC values for these time points in the training set were 0.855, 0.887, 0.943, and 0.932 (**Figure 6A**), while in the internal validation set, they were 0.781, 0.788, 0.897, and 0.872 (**Figure 6B**). The change in AUC values over time for both the training and internal validation sets is illustrated in **Figures 6C**, **D**. Furthermore, calibration curves for predicting OS at each time point were plotted, including the training set (**Figures 7A–D**) and the internal validation set (**Figures 7E–H**).

**Figure 8:**
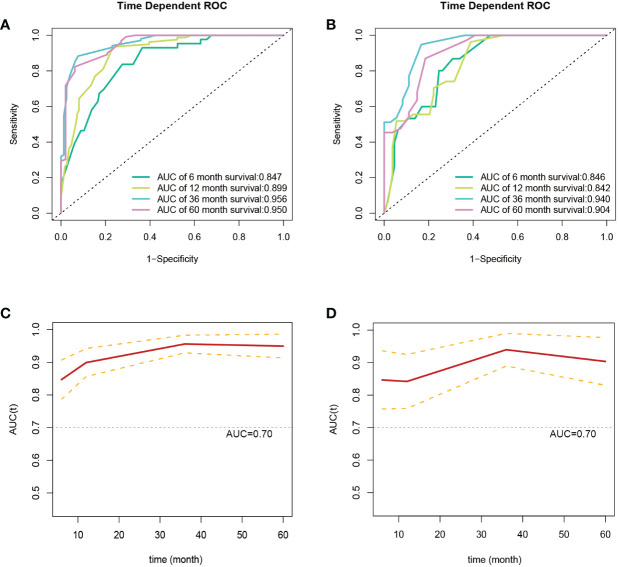
ROC and AUC curves for predicting CSS in GB-NENs patients. **(A)** ROC curve for the training set; **(B)** ROC curve for the internal validation set; **(C)** AUC curve for the training set; **(D)** AUC curve for the internal validation set. ROC, receiver operating characteristic; AUC, area under the receiver operating characteristic curve; CSS, cancer-specific survival; GB-NENs, gallbladder neuroendocrine neoplasms.

**Figure 9:**
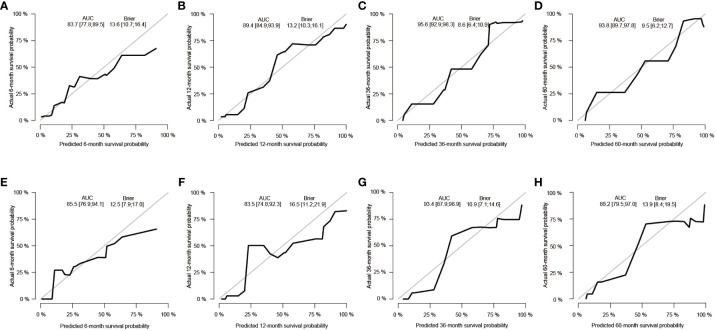
Calibration curves for predicting CSS in GB-NENs patients. **(A–D)** calibration curves for 6, 12, 36, and 60 months in the training set; **(E–H)** calibration curves for 6, 12, 36, and 60 months in the internal validation set. CSS, cancer-specific survival; GB-NENs, gallbladder neuroendocrine neoplasms.

Subsequently, using data from the training set, we created a CSS predictive nomogram for GB-NENs. It included four prognostic factors, age, histology type, tumor size, and surgery, all of which were encompassed within the OS prognostic factors. The results are presented in **Figure 5B**. In the internal validation, the C-index of the nomogram was calculated to be 0.831 (95% CI, 0.793-0.868), indicating good discriminative ability. Similar to the OS model, time-ROC analysis was performed to evaluate the predictive capability at four different time points (6 months, 12 months, 36 months, and 60 months). The AUC values in the training set for these time points were 0.847, 0.899, 0.956, and 0.950 (**Figure 8A**), while in the internal validation set, they were 0.846, 0.842, 0.940, and 0.904 (**Figure 8B**). The change in AUC values over time for both the training and internal validation sets is presented in **Figures 8C**, **D**. Lastly, calibration curves for each time point were plotted for both the training set (**Figures 9A–D**) and the internal validation set (**Figures 9E–H**), resulting in satisfactory outcomes for CSS.

### 3.5 Clinical application of the nomogram

**Figure 10:**
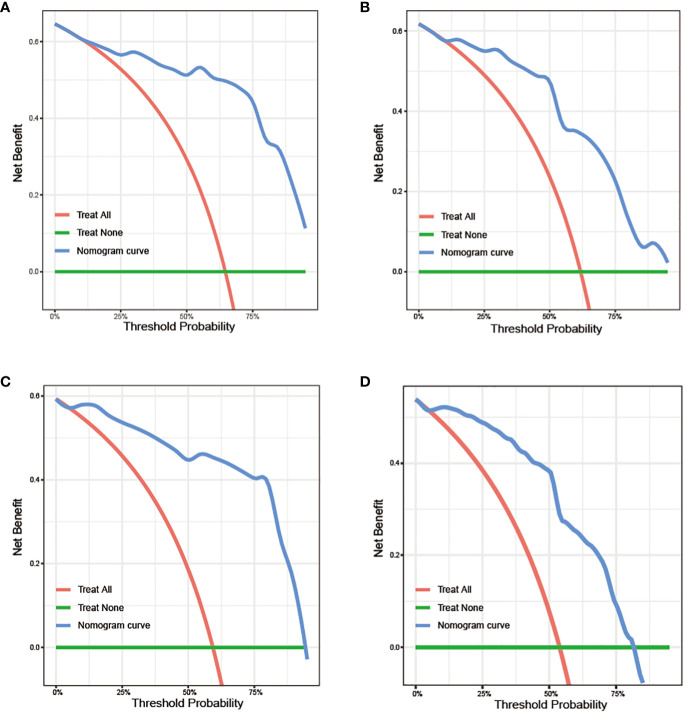
DCA curves for predicting OS and CSS in GB-NENs patients. **(A)** training set and **(B)** internal validation set for OS; **(C)** training set and **(D)** internal validation set for CSS. DCA, decision curve analysis; OS, overall survival; CSS, cancer-specific survival; GB-NENs, gallbladder neuroendocrine neoplasms.

To assess the clinical utility of the nomograms, we first generated DCA curves, which included the performance of GB-NENs OS in the training set and internal validation set (**Figures 10A**, **B**), as well as GB-NENs CSS in the training set and validation set (**Figures 10C**, **D**). These DCA curves demonstrated favorable clinical benefits within various intervals.

**Figure 11:**
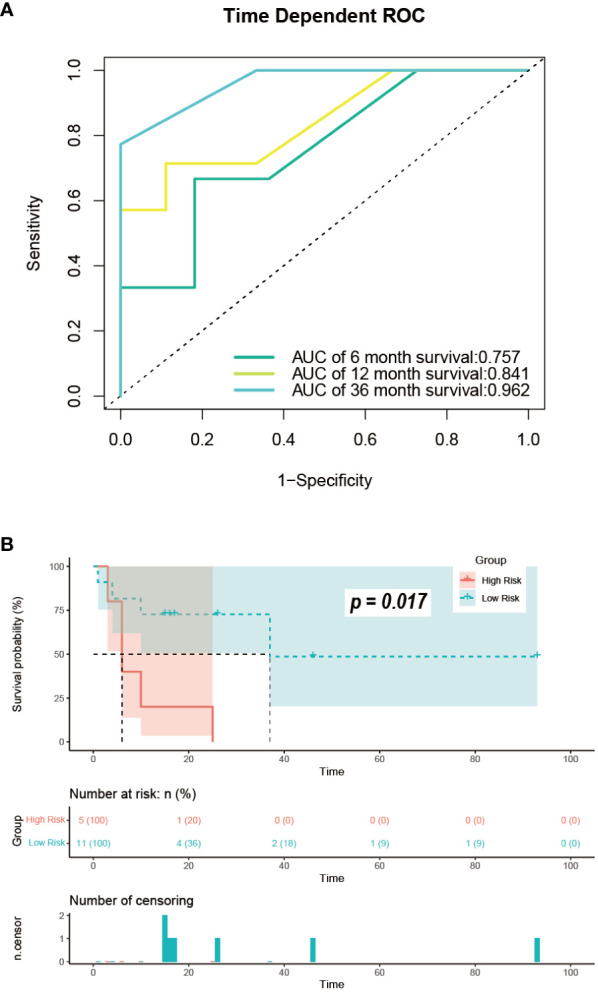
Performance in the external validation set. **(A)** ROC curves for predicting OS at 6, 12, and 36 months; **(B)** risk stratification. ROC, receiver operating characteristic; OS, overall survival.

We obtained data from 16 GB-NENs patients at the Pathological Diagnosis Center of Jilin University First Hospital (see **Table 3**). Due to the rarity of this disease and limited follow-up time, we validated the 6-month, 12-month, and 36-month OS. AUC values of 0.758, 0.841, and 0.962 were obtained (**Figure 11A**). The AUC values at these three time points displayed similar trends to the performance in the training and internal validation sets, highlighting the strong clinical applicability of our established model.

**Table 3:** Clinical characteristics of the external validation set in GB-NENs.

Characteristic	Patients	%
All	16	100
Histology type		
NETs	4	25.0
NECs/MANECs	12	75.0
Gender		
Female	6	37.5
Male	10	62.5
Race		
White	0	0
Black	0	0
Other	16	100
Age, y		
≤60	3	18.8
60~80	10	62.5
>80	3	18.8
Marital status		
Single	1	6.2
Married	11	68.8
Other	4	25.0
Year of diagnosis		
<2010	0	0
≥2010	16	100
First primary malignancy		
No	3	18.8
Yes	13	81.2
Tumor size, mm		
≤50	11	68.8
>50	4	25.0
Unknown	1	6.2
Pathological grade		
I/II	0	0
III/IV	0	0
unknown	16	100
Surgery on primary site		
No/Unkown	5	31.2
Yes	11	68.8
Lymph node surgery		
No/Unkown	11	68.8
Yes	5	31.2
Radiation		
No/Unkown	13	81.2
Yes	3	18.8
Chemotherapy		
No/Unkown	7	43.8
Yes	9	56.2

NETs, neuroendocrine tumors; NECs, neuroendocrine carcinomas; MANECs, mixed adenoneuroendocrine carcinomas.

### 3.6 Risk classification system

**Figure 12:**
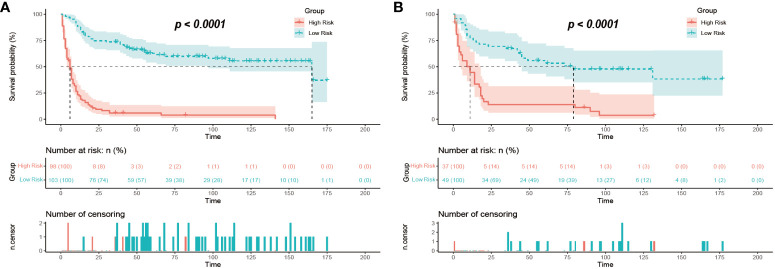
Risk stratification of mortality in GB-NENs patients. **(A)** training set; **(B)** internal validation set. GB-NENs, gallbladder neuroendocrine neoplasms.

For the GB-NENs OS predictive nomogram we developed, we calculated the scores for each patient in the training set. Based on the risk score and survival outcomes, we determined the optimal cut-off value for the model as 166.5, then divided the patients into high-risk and low-risk groups using this optimal cut-off value. The Kaplan-Meier curves displayed that the OS model effectively assessed the patients’ prognosis. The median OS time for the high-risk group was 6 months, and for the low-risk group, it was 165 months (p < 0.001). This optimal cut-off value also demonstrated good predictive performance in the validation sets. In the internal validation set, the median OS time for the two groups was 11 months and 79 months (p < 0.001), as shown in **Figures 12A**, **B**. In the external validation set, it was 6 months and 37 months (p < 0.001), as illustrated in **Figure 11B**.

## 4 Discussion

GB-NENs are relatively rare gallbladder lesions that exhibit unique characteristics, often encountered in case reports and clinical studies with limited sample sizes (7, 17, 27). The origin of GB-NENs remains unclear. NENs primarily occur in the rectum, ileum, and appendix, where hormone-producing cells called amine precursor uptake and decarboxylation (APUD) cells are present (28). However, these cells are lacking in the mucosa of the gallbladder. Several hypotheses have been proposed to explain the origin of GB-NENs, including metaplasia of gallbladder epithelium to intestinal or gastric epithelium, pluripotent cell origin, and ADCs transformation, but none have been confirmed (14, 28). Notably, the prognosis of GB-NENs is significantly worse compared to NENs from other abdominal organs in contemporary studies (16).

Currently, diagnosis relies on pathological and immunohistochemical analyses. According to the, 2019 World Health Organization classification of gastroenteropancreatic NENs, these neoplasms are divided into three grades based on mitotic rate and Ki-67 index: Well-differentiated neuroendocrine tumors (NETs), poorly-differentiated neuroendocrine carcinomas (NECs), and mixed neuroendocrine non-neuroendocrine neoplasms (MiNENs) (29). In contrast to NENs in the pancreas and appendix, which predominantly belong to the G1/G2 grades, the majority of GB-NENs are found to be malignant neuroendocrine carcinomas, closely associated with a poorer prognosis (30, 31). Immunohistochemical markers such as Ki-67, chromogranin A (CgA), synaptophysin (Syn), and neuron specific enolase (NSE) play a crucial role in the diagnosis of NENs (17).

ADC is the most common histology type among gallbladder malignant tumors, constituting 76-90% of all malignancies, whereas GB-NENs account for only about 2.1% (8). While GB-NENs exhibit a higher malignancy rate than GB-ADCs. Upon reviewing existing research, considerable controversy arises regarding the prognostic comparison between GB-NENs and GB-ADCs. In recent years, the prevailing viewpoint among scholars leans towards a poorer prognosis for GB-NENs (14, 27, 32, 33). To obtain more compelling conclusions, Bae et al. (34) and Yan et al. (7) employed PSM to balance differences between the two groups. Ultimately, they found that patients with GB-ADCs have significantly longer OS than GB-NECs patients. Conversely, Yun et al., in a study involving 4 GB-NENs cases and 38 GB-ADCs cases, concluded that GB-NENs had a more favorable prognosis, although statistical significance was not attained (19). Furthermore, a multicenter retrospective study in, 2019 discovered that the postoperative prognosis for GB-NENs was superior (16). However, no further in-depth investigation or explanation of the reasons behind this phenomenon was conducted. Regarding two recent high-quality cohort studies, Hu et al. (18) and Do et al. (17) reported that GB-NECs and GB-ADCs patients have similar prognoses. It is not difficult to observe that these studies were constrained by small sample sizes or incomplete research, inherently introducing significant errors and lack of representativeness. In our study, we included a sufficient number of GB-NENs cases. Our findings indicated that both the OS and CSS of GB-NENs patients were superior to GB-ADCs before and after PSM.

In terms of clinical outcomes, We calculated a median OS time of 18 months for GB-NENs, coinciding with the reported range of 12-20 months in recent research studies (7, 17, 27, 32, 34–37). The 1, 3, and 5-year OS rates for GB-NENs were 58.2%, 41.5%, and 35.8%, respectively, higher than those of GB-ADCs, which were 46.1%, 23.0%, and 17.0%, respectively. Studies by Lee et al. (27) and Yan et al. (7) reported 1, 2, and 3-year OS rates for GB-NEC cases in East Asia, which were lower than those for concurrent ADCs. In a recent multicenter retrospective study led by Wang et al. (31), GB-NENs demonstrated 1, 3, and 5-year survival rates of 59%, 33%, and 29%, respectively. It is worth mentioning that this study included 8.3% of NETs cases. In comparison with our results, these findings collectively indicate a worse prognosis, despite improvement compared to patients included in earlier original studies (8, 33, 38).

The discrepancies in these conclusions can be attributed to several factors. Firstly, the difference in the histology types of cases included is a crucial factor. GB-NENs are divided into NETs, NECs, and MiNENs, with substantial differences in prognosis among these categories (35). Our study included a relatively higher proportion of NETs patients (29.6%), which has a better overall prognosis. Secondly, variations in treatment strategies play a significant role (16). The lack of standardized management for this disease leads to considerable diversity in clinical practices, contributing to differences in prognosis. Lastly, geographical differences may also be a contributing factor. Our study involved cases from various regions of the United States, whereas most of the aforementioned research was conducted in the Asia-Pacific region, where variations in disease incidence and treatment understanding might lead to different outcomes.

Furthermore, we constructed two nomograms to estimate OS and CSS for GB-NENs. We identified three clinical factors (older age, poorer pathological grade, and larger tumor size) as independent risk factors for prognosis, while first primary malignancy and surgery on primary site were independent protective factors. In our study, the TNM stage, typically associated with clinical prognosis, was excluded due to excessive missing data. Nevertheless, the predictive model demonstrated excellent performance in terms of survival, with AUC values reaching 0.847, 0.899, 0.956, and 0.950 for predicting 6, 12, 36, and 60-month OS in the training set. In the internal validation set, AUC values were 0.847, 0.842, 0.940, and 0.904, and in the external validation set, they were 0.758, 0.841, and 0.962 (60-month value not available). The model showed superior performance in predicting long-term prognosis. Therefore, the absence of these variables did not significantly affect the overall model performance, suggesting a limited impact on prognosis compared to other malignancies.

Age, as a vital prognostic factor, has been observed in previous studies of NENs in other sites (39–42). Aging increases the likelihood of oncogene mutations and, in combination with comorbid chronic illnesses, leads to a diminished ability to resist surgical stress, resulting in poorer survival rates for elderly GB-NENs patients (39, 40, 43). Tumor size and pathological grade are indicators of more extensive tumor invasion and greater aggressiveness, which have been previously demonstrated in research (36, 44, 45). In addition, our study emphasized the importance of surgery as a crucial factor affecting patient prognosis. The roles of radiation therapy and chemotherapy were not evident. As with other cancer types, surgical treatment for GB-NENs is widely accepted among researchers, and related studies have demonstrated the importance of surgery in improving survival (17, 46, 47). The significance of postoperative adjuvant chemotherapy in the prognosis of GB-NENs is gradually being confirmed by other relevant research (14, 48). Studies indicate that combined surgery and adjuvant chemotherapy significantly enhance both short-term and long-term survival, and when radical resection is not feasible, adjuvant chemotherapy becomes the treatment of choice (17).

Moreover, early diagnosis is an urgent area for improvement in changing patient prognosis through surgery. Due to the lack of early symptoms, GB-NENs are often diagnosed at advanced stages, sometimes with distant organ metastasis, leading to a poor prognosis (16). Currently, diagnosis primarily relies on pathological examinations and immunohistochemical analyses. In clinical practice, Computed Tomography (CT) is still used as the main tool to evaluate primary gallbladder cancer (49), Kim et al. (32) extracted the CT features of GB-NENs and GB-ADCs, and found that masses possessing clearer borders and stronger enhancement can be used as imaging features of GB-NENs. Further, Bae et al. attributed the features of Magnetic Resonance Imaging (MRI) of GB-NENs: distinct borders, intact overlying mucosa, and thicker margins, etc., and these preoperative tests are expected to play a crucial role in the diagnosis of GB-NENs. In addition to differentiation from GB-ADCs, focal nodular hyperplasia, hypervascular metastases and hepatocellular carcinoma infiltration must also be considered (50).

Our study on GB-NENs assessed the differences in prognosis with GB-ADCs and explored various factors associated with prognosis. We established two nomograms that accurately predict prognosis, demonstrating promising results with potential clinical implications. However, it is essential to acknowledge the following limitations in our research. Firstly, our study’s retrospective nature and reliance on a public database introduced selection bias. While the nomograms yielded favorable results, the limited number of cases in the external validation set hindered a comprehensive evaluation of its performance and stability. Secondly, several clinically important variables, such as T, N, and M staging, were excluded from the analysis due to extensive missing data. Even when considering variables like tumor size and grade, substantial data gaps made PSM and modeling unavoidably introduce some degree of bias, and the interpretation of the results should be approached with caution. Finally, with regard to treatment, we were unable to access a sufficient number of cases with detailed information on surgical procedures and specific adjuvant treatment regimens. Consequently, we could only broadly categorize treatments. This, to some extent, impacted our ability to investigate the relationship between treatment modalities and prognosis.

## 5 Conclusion

In this study, we observed that the overall prognosis of GB-NENs patients is superior to that of GB-ADCs patients. Even after balancing the baseline clinical characteristics of both groups through PSM, we obtained consistent and statistically significant results. Furthermore, we identified age, histology type, first primary malignancy, tumor size, and surgery as independent prognostic factors for GB-NENs. Based on these factors, we developed two predictive nomograms to estimate individual survival rates for GB-NENs patients. These models demonstrated promising clinical utility and can be applied in GB-NENs clinical practice.

## Data availability statement

The original contributions presented in the study are included in the article/supplementary material. Further inquiries can be directed to the corresponding author.

## Ethics statement

Ethical approval was not required for the study involving humans in accordance with the local legislation and institutional requirements. Written informed consent to participate in this study was not required from the participants or the participants’ legal guardians/next of kin in accordance with the national legislation and the institutional requirements.

## Author contributions

Z-HZ: Conceptualization, Data curation, Formal analysis, Methodology, Software, Validation, Visualization, Writing – original draft, Writing – review & editing. YH: Conceptualization, Data curation, Formal analysis, Methodology, Software, Validation, Visualization, Writing – review & editing. CJ: Data curation, Investigation, Methodology, Project administration, Resources, Supervision, Writing – review & editing. G-YL: Conceptualization, Project administration, Resources, Supervision, Writing – review & editing. MW: Conceptualization, Funding acquisition, Investigation, Project administration, Resources, Supervision, Writing – review & editing.
